# Supplementary figures and images for: Ketone-Dependent Restoration of Autophagy and Mitochondrial Quality Control Through VPS35 in a Drosophila Model of C99-Induced Neurodegeneration
Source: Cells. 2026 Jun 15;15(12):1082. doi: 10.3390/cells15121082 (PMC13297195; doi:10.3390/cells15121082)

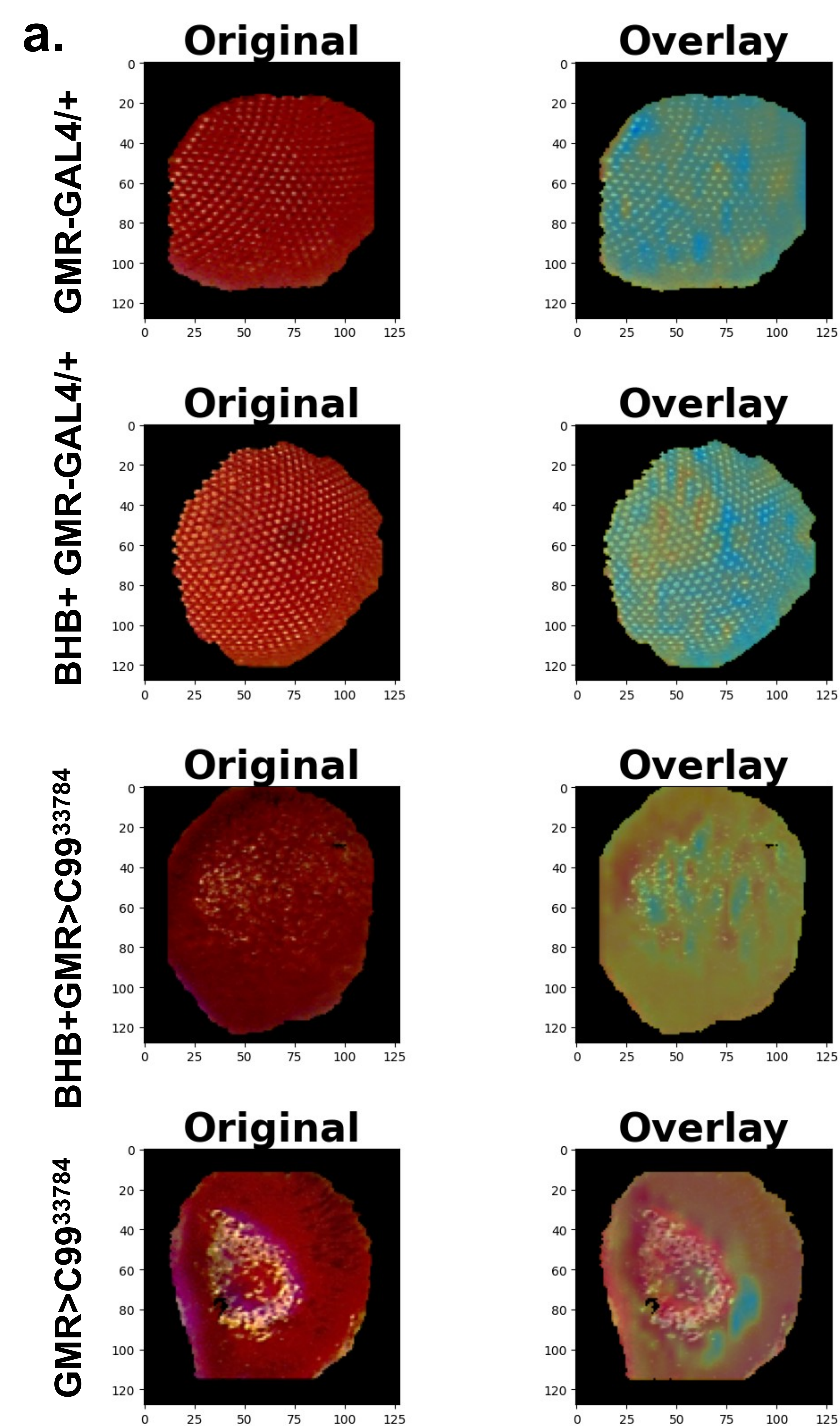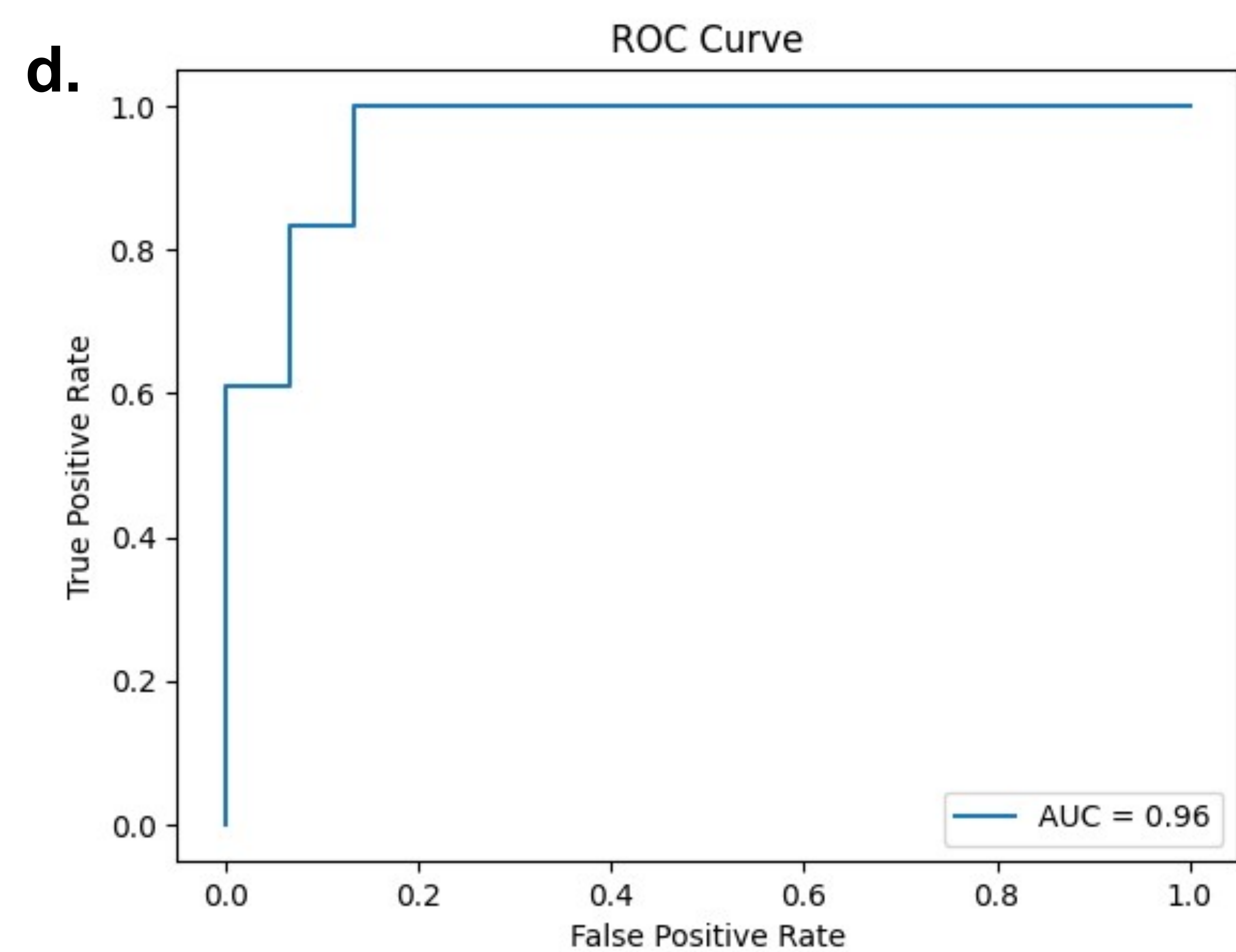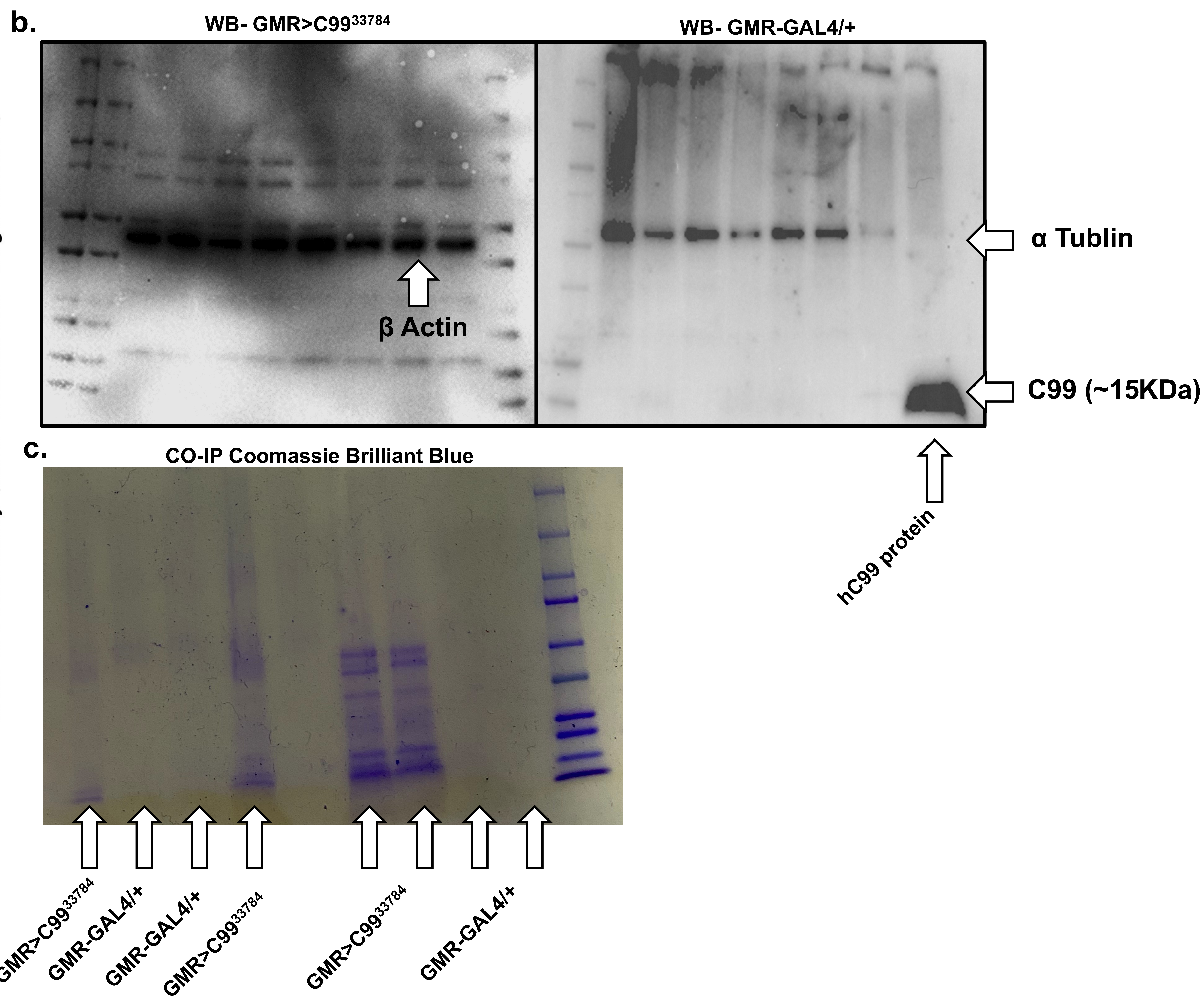

Supplement: Supplementary file 1 [file cells-15-01082-s001.zip › Figure S1.pdf]
